# Healthcare Crisis in Bangladesh during the COVID-19 Pandemic

**DOI:** 10.4269/ajtmh.20-0826

**Published:** 2020-08-20

**Authors:** Md. Sayeed Al-Zaman

**Affiliations:** Department of Journalism and Media Studies, Jahangirnagar University, Dhaka, Bangladesh

## Abstract

The COVID-19 pandemic has had a severe impact worldwide. Developed countries, such as the United States, United Kingdom, Italy, and Spain, had their highly efficient medical infrastructure greatly stressed and suffered from high death tolls. Similarly, Bangladesh, a poverty-stricken South Asian country, is losing its battle against the pandemic, but mainly because of its incompetent healthcare system. The casualties are escalating and public sufferings are becoming unimaginable. On this backdrop, this perspective piece discusses the healthcare crisis in Bangladesh during the pandemic. This article also identifies three responsible issues for the country’s deteriorating health care: 1) poor governance and increased corruption, 2) inadequate healthcare facilities, and 3) weak public health communication.

Surprisingly, whereas many developed countries, including the United States, the United Kingdom, Italy, and Spain, have suffered greatly during the COVID-19 pandemic even with their highly efficient healthcare systems, many less developed countries with inadequate healthcare systems are surviving the crisis more easily.^1^ Where is the magic? Unfortunately, no magic is there. Rather, behind this mysterious façade, the reality tells a different tale. This pandemic reveals the incompetent health care of many less developed countries engulfed by intense corruption, and Bangladesh is one of them. As one of the world’s most densely populated countries (1,115 people/km^2^) with 21.8% of people living below the poverty line,^2^ Bangladesh has a healthcare system that lacks reliability, responsiveness, and empathy, and that has already been proved inadequate to deliver proper health care to the public on many occasions.^3,4^ Moreover, medical facilities are concentrated in urban areas that create a *healthcare divide* depriving rural areas.^3,5^ Amid such a situation, the COVID-19 pandemic reveals many loopholes in the healthcare system that can be summarized under three themes: 1) poor governance and increased corruption, 2) inadequate healthcare facilities, and 3) weak public health communication.

After detecting the first COVID-19 case on 8 March, in the following 2 weeks, more than 0.63 million overseas workers entered the country without proper screening, facilitating community transmission.^6^ Furthermore, a few of the government’s delayed decisions were found less effective that worsened the pandemic situation. For example, the government declared a general holiday for 10 days from March 23, 2020 to April 2, 2020 without restricting transportations and public movement. As a result, a crowd of more than 12 million people left Dhaka immediately after the notice that expedited the community transmission.^7^ Moreover, none of the two existing laws, Disaster Management Act 2012 and Infectious Disease (Prevention, Control, and Elimination) Act 2018, were brought into action properly to control such massive mass mobilizations and gatherings.^6^ In such a situation, many private medicals around the country were either unwilling or abstained from treating COVID-19 patients, and thus healthcare denial intensified.^8,9^ A survey found that 77.3% of patients receive healthcare facilities from private medicals.^6^ Consequently, a large share of the population suffered, and many of them died without proper medical supports.^10^ Improper synchronization among the responsible bodies, the Ministry of Health and Family Welfare (MoHFW), the Directorate General of Health Services, and test laboratories’ executives and workers, could be a reason for such mismanagements.^11^

With the deteriorating COVID-19 situation in Bangladesh, corruption surged. As a great initiative, Prime Minister Sheikh Hasina promptly declared 19 incentive packages of USD 12.13 billion to ameliorate people’s sufferings from the pandemic.^6^ But it is a matter of concern how effective these measures could be amid the intensifying nationwide corruption. Whereas mismanagements were paralyzing the health sector, increased corruption worsened the situation to a greater degree.^6^ The media reported 218 relief-related corruption incidents from March 10, 2020 to June 15, 2020, and most of the convicts were the government officials, public representatives, and ruling party leaders and activists.^12^ They either expropriated the public’s relief or counterfeited the budgets of medical equipment and health services.^6,12^ In July, a hospital owner along with a few responsible persons was convicted for trading with fake COVID-19 test certificates (for more fake COVID-19 test news, see https://tinyurl.com/y23abr3o). More investigations reveal similar incidents, including the case of two doctors who threw thousands of patients’ samples in the garbage and provided them imaginary results (for a few notable examples, see Table 1).^13,14^ Despite the rampant irregularities and corruptions, a government promulgation on August 5, 2020 restricted law enforcement’s investigations in hospitals: specialists suggest that it would exacerbate health-related corruption.^15^

**Table 1:** A few notable examples of corruption in the health sector during the COVID-19 pandemic

Corruption	Specific details*
Medical equipment for Tajuddin Medical College	The proposed expenditure was USD 20.70 million that was at least 10 times higher than the actual expenditure. Also, the products were of poor quality.
Website development	The proposed expenditure was USD 1.18 million, and the original expenditure was USD 9438.
Computer software	The proposed expenditure was USD 6.49 million, and the original expenditure was USD 0.17 million.
Safety goggles	The proposed expenditure was USD 59/piece, and the market price was USD 12/piece.
Audio clips	The proposed expenditure was USD 1.36 million, which was unevenly higher than the market price.
Personal protective equipment	The proposed expenditure was USD 52/piece, and the original market price was less than USD 23/piece.
Five hundred physicians’ food and living cost for 1 month	The proposed expenditure was USD 23.60 billion, and the original expenditure could be far less than the proposed expenditure.
Regent Hospital scam	Hospital owned by Shahed (42) carried out 10,500 COVID-19 tests, of which 6,300 were fake reports.
JKG Health Care scam	Approved by Directorate General of Health Services, JKG Health Care set up 44 booths for sample collection. Each day, the workers collected 500 samples. Providing fake test reports to the public, they earned USD 0.94 million.

Sources: The information is collected from media reports published in March 10, 2020–July 30, 2020.

* The explanations and estimations are based on both the media reports and the current market price. The amounts are converted from Bangladeshi Taka to USD according to the exchange rate of August 1, 2020.

Healthcare preparation and capacity against COVID-19 might explain the pandemic situation in Bangladesh more precisely. The coronavirus testing rate in Bangladesh (0.34%) is the second lowest in South Asia only after Afghanistan, a war-torn nation (Table 2).^6^ Its main reasons could be the limited number of testing laboratories (56 laboratories) and kits, and their uneven distributions across the country, expensive coronavirus tests in private medicals (USD 50–60/test), the fewer number of medical workers, and unregulated testing system (elites get preferences).^1,6,11^ Moreover, many testing kits were preserved by corrupt businessmen to initiate an artificial crisis.^16^ Recently, the MoHFW imposed a fee on COVID-19 test in state-run laboratories too that dramatically reduced the average per day tests.^17^ Thanks to the lower test rate, 1,010 people died undiagnosed with coronavirus symptoms from March 2020 to May 2020.^18^ It hints about the possible discrepancies in the coronavirus’s official statistics of Bangladesh. Medical facilities, such as beds, intensive care units, and ventilators, are far fewer than the required amount in both government and private hospitals.^19^ Therefore, to manage a seat in the country’s finest hospitals, patients often need to have connections. Also, many patients prefer to remain at home fearing maltreatment in hospitals.^10^ A report reveals that 79% of patients stay at home and get treatment over the phone.^20^ In Bangladesh, only 3.05 physicians and 1.07 nurses serve every 10,000 people on average,^5^ which is insufficient for the pandemic situation. Moreover, medical workers were provided lower quality medical equipment, such as masks and personal protective equipment. Consequently, many doctors got infected and some died, making the doctors’ mortality rate of Bangladesh highest in the world.^21^

**Table 2:** Reported tests per 1,000 people in South Asian countries

Rank	Country	Test per 1,000	Percent
1	Maldives	69.4	6.9
2	Bhutan	30.3	3.0
3	India	4.9	0.5
4	Pakistan	4.8	0.5
5	Nepal	4.6	0.5
6	Sri Lanka	4.2	0.4
7	Bangladesh	3.4	0.3
8	Afghanistan	1.6	0.2

Sources: Johns Hopkins Corona Resource Center, the World Bank, and Our World in Data.

Inadequate information flow and communication networks make the healthcare system more vulnerable and incompetent. Because of health-related uncertainty, information scarcity, the absence of reliable information sources, and defected flow of reliable information amid the pandemic, rumors became prevalent. In such a situation, national media outlets failed to successfully deliver reliable information to a large number of audiences, letting the more personalized and Internet-based media occupy the communication space. As a result, around 200 COVID-19–related online rumors spread across the country from March 2020 to July 2020 (for the complete list of rumors, see http://bdfactcheck.com, a nonprofit award-winning fact-checking website of Bangladesh). As a timely step, the government started detaining rumor-producers and rumor-spreaders to reduce the COVID-19 crisis to some degree. However, along with the perpetrators, as many human rights activists and organizations believe thanks to a few recent incidents, political dissidents and the government’s critics may be suppressed.^22,23^ Meanwhile, government officials are ordered “not to like, share or comment on social media posts” that criticize the government’s policies.^22^ These steps may breed fear among the online communities and hamper the positive health-related communication.

In June 2020 after a visit, a team of Chinese physicians expressed their concerns about Bangladesh’s disorganized health sector: this article already discussed the key selected discrepancies.^24^ This situation may be controlled by taking a few steps. First, corruption in the health sector is mandatory as this will help improve the proper utilization of allocated resources. Second, more tests should be conducted to identify the infected persons to provide them better treatment. Third, hospitals should be well equipped with updated and efficient medical supplies such as oxygen and medications to provide supportive treatment for COVID-19. Fourth, doctors and other medical workers must be protected from infection. Moreover, infected doctors and nurses could be super-spreaders of the virus. Fifth, thanks to higher population density and lower health awareness, social distancing in public spaces is virtually impossible in Bangladesh. Therefore, some sort of strict regulations for such spaces may be imposed. Sixth, healthy information flow is a must for the current COVID-19 situation in Bangladesh to reduce the health-related confusions and uncertainties. Battling the COVID-19 pandemic without organized strategies and an effective healthcare system would be like an attempt to kill a lion with bare hands.
